# Timing of Anticonvulsant Administration in Status Epilepticus

**DOI:** 10.15844/pedneurbriefs-29-6-2

**Published:** 2015-06

**Authors:** Juan A. Piantino

**Affiliations:** 1 Department of Pediatrics, Section in Child Neurology – Epilepsy, Oregon Health and Science University, Portland, OR

**Keywords:** Pediatrics, Status Epilepticus, Anticonvulsant

## Abstract

Investigators from the Pediatric Status Epilepticus Research Group studied the time elapsed from onset of pediatric convulsive status epilepticus (SE) to administration of antiepileptic drugs (AED).

Investigators from the Pediatric Status Epilepticus Research Group studied the time elapsed from onset of pediatric convulsive status epilepticus (SE) to administration of antiepileptic drugs (AED). This prospective observational cohort study enrolled pediatric patients (1 month–21 years) with convulsive SE. In order to study timing of AED administration during all stages of SE, the investigators restricted their study population to patients who failed 2 or more AED classes or needed continuous infusions to terminate convulsive SE. Eighty-one patients were enrolled (44 male) with a median age of 3.6 years. The first, second, and third AED doses were administered at a median (p25–p75) time of 28 (6–67) minutes, 40 (20– 85) minutes, and 59 (30–120) minutes after SE onset. Considering AED classes, the initial AED was a benzodiazepine in 78 (96.3%) patients and 2 (2–3) doses of benzodiazepines were administered before switching to non-benzodiazepine AEDs. The first and second doses of non-benzodiazepine AEDs were administered at 69 (40–120) minutes and 120 (75–296) minutes. In the 64 patients with out-of-hospital SE onset, 40 (62.5%) patients did not receive any AED before hospital arrival. In the hospital setting, the first and second in-hospital AED doses were given at 8 (5–15) minutes and 16 (10–40) minutes after SE onset (for patients with in-hospital SE onset) or after hospital arrival (for patients with out-of-hospital SE onset). The authors concluded that the time elapsed from SE onset to AED administration and escalation from one class of AED to another is delayed, both in the pre-hospital and in-hospital settings. [1]

COMMENTARY. Current status epilepticus (SE) treatment protocols recommend a timely administration of AED doses and a rapid escalation between different classes of AED's [2]. The rationale for this recommendation includes results from clinical studies suggesting better seizure control and reduction of brain injury with earlier AED administration [3]. Results from animal models showed that prolonged SE causes brain damage [4], and the response to benzodiazepines decreases with seizure duration [5, 6]. While these data suggest the importance of rapid AED administration in SE, there is limited literature on the timeliness of AED administration in clinical practice. Additionally, there are no series that have systematically studied the time of AED administration at all stages of SE treatment. This study addresses these gaps in knowledge. In the pre-hospital setting, more than half of the patients did not receive any AED until hospital arrival. Interestingly, lack of pre-hospital AED administration also occurred in patients with a prior diagnosis of epilepsy or prior SE episode, a group in whom a plan should have been devised. Although the study does not address what causes delays in drug administration, the authors identified several areas for improvement including earlier detection and treatment of seizures, more widespread use of home rescue BZD's, and rapid escalation of AED treatment or early poly-pharmacotherapy. Their results support the implementation of policies that optimize timing and escalation of AED administration for seizures at the family, EMS, and hospital levels.
